# Dynamic serum alkaline phosphatase is an indicator of overall survival in pancreatic cancer

**DOI:** 10.1186/s12885-019-6004-7

**Published:** 2019-08-07

**Authors:** Yuanyuan Xiao, Jian Lu, Wei Chang, Ying Chen, Xiaomei Li, Dehui Li, Chuanzhi Xu, Haijun Yang

**Affiliations:** 1 School of Public Health, Kunming Medical University, Kunming, 1168 West Chunrong Road, Kunming, Yunnan China; 2 The Third Affiliated Hospital, Kunming Medical University, 519 Kunzhou Road, Kunming, Yunnan China

**Keywords:** Alkaline phosphatase, Pancreatic ductal adenocarcinoma, Survival

## Abstract

**Background:**

The prognostic role of serum alkaline phosphatase (ALP) has been found in several kinds of solid malignant tumor, but has never been extensively discussed in pancreatic cancer, especially through the application of dynamic survival model which incorporates the varying nature of ALP measurements.

**Methods:**

We conducted a retrospective study which successfully collected 551 histopathologically confirmed pancreatic ductal adenocarcinoma (PDAC) patients from a cancer specialized hospital in southwest China. The association between variant ALP which measured during the whole survival period and the overall survival (OS) of PDAC patients was evaluated by using dynamic Anderson-Gill (AG) model. Exhaustive sensitivity analysis was performed by adopting continuous cut-offs of ALP.

**Results:**

After adjusted for possible confounding of serum albumin, total bilirubin and leukocyte counts, AG model revealed that, serum ALP during the survival period was nonlinearly associated with the OS of PDAC: for resected patients, compared with those whose ALP results ranged within the first quartile (<P_25_), patients whose ALP measurements belonged to the second (P_25_-P_50_), the third (P_50_-P_75_), and the forth (>P_75_) quartiles were observed 1.14 (95% CI: 0.29–4.56), 3.93 (95% CI: 1.23–12.60), 3.87 (95% CI: 1.32–11.36) folds of death hazard; whereas in un-resected PDAC patients, the hazard ratios (HRs) were 1.15 (95% CI: 0.79–1.68), 1.92 (95% CI: 1.32–2.78), and 1.97 (95% CI: 1.30–2.98), respectively. Sensitivity analysis revealed that, for both resected and un-resected patients, the results of AG model were robust with regard to various cut-offs of ALP, and an increased ALP was in general associated with significantly increased hazard of death.

**Conclusion:**

Serum ALP during the survival period was significantly associated with the OS of PDAC patients, especially for resected early stage PDAC patients. Future studies with expanded sample size and refined prospective design should be implemented to corroborate our major findings. Besides, the underlying mechanism for this possible hazardous role of ALP should also be investigated.

## Background

Pancreatic cancer (PC) remains one of the most lethal malignant tumors. It has been estimated that, in the year of 2015, PC caused 411, 600 deaths, makes it the seventh most common cause of death from cancer globally [1]. According to surveillance data which collected from a nationally representative sample accounted for 13% of the entire population, PC related mortality was also ranked the 7th among all cancers in China [2]. The many pathological types of PC can be mainly categorized into two groups: exocrine cancers and neuroendocrine cancers. The exocrine PC is dominated by pancreatic ductal adenocarcinoma (PDAC), which accounted for nearly 85% of all PC cases [3].

Lack of specific clinical symptoms or manifestations in the early phase of the disease, as well as the extremely slow progress in effective chemotherapy drugs are the main culprits for dismal outcome of PC patients: only less than 20% of PC patients can be diagnosed with a localized disease which can be treated with surgical operation of curative intention [4], and in the last decade, the improvement in chemotherapy regimen only increased the survival length by a few months [5]. Currently, the median survival length for all diagnosed PC patients is 3–6 months, and the 5-year survival rate is generally less than 5% [6–9].

Under this circumstance, an expanded search on indicators which are of prognostic interest of PC is necessary and imperative. The easy availability and usually low cost made common blood indicators ideal biomarkers of study interest in the field of cancer epidemiology. Since the year 2000, the association between blood indicators and cancer survival has been expansively investigated, however, compared with other common types of cancers, the studies regarding to PC is still severely deficient. Alkaline phosphatase (ALP) is a homodimeric enzyme which main function is removing phosphate groups. In humans, ALP is expressed in all tissues or organs, but is particularly concentrated in liver, bile duct, kidney and bones. During the past several years, researchers have reported that the elevation of serum ALP was prominently related to a less optimistic prognosis of prostate cancer [10–12], colorectal cancer [13], triple negative breast cancer [14], nasopharyngeal cancer [15] and esophageal cancer [16]. Nevertheless, the association between serum ALP which measured at eventful moments, such as upon diagnosis, before or after curative resection, and PC survival has never been intensively discussed. Besides, during the whole survival period, ALP measurements of PC patients will inevitably vary from test to test, therefore, dynamic survival models which can further adjust for this time-varying nature of ALP should also be used to generate a more credible conclusion.

With regard to the aforementioned deficiencies, in this study, we intend to explore the prognostic value of serum ALP among a large group of Chinese PC patients. Both static Cox proportional hazards model and a seldom used dynamic survival model will be applied to fully evaluate the association between ALP and the overall survival (OS) of PC.

## Methods

### Study design

The patients we studied were histopathologically confirmed PDAC patients who were newly diagnosed between January 1, 2015 and December 31, 2016 at the Third Affiliated Hospital of Kunming Medical University. Relevant information was retrospectively reviewed and extracted from the hospital information system (HIS), which include demographic characteristics (gender, age), operation details (modality of operation), chemotherapy regimens, test results of serum ALP and three other possibly related blood indicators (albumin, total bilirubin, leukocyte count). Only diagnosed patients with complete information were included. Because our major focus was to evaluate the association between varying ALP and the OS of PC, all test results of ALP, albumin, total bilirubin, and leukocyte count were collected between the date of diagnosis or curative operation and January 1, 2018, a predetermined deadline of the study. Death information was ascertained for each PDAC patients subsequently, through searching the unique personal ID in Chinese Residents Death Registration System, a death surveillance system which covers the whole mainland Chinese population.

### Variables and definitions

In this study we included both early stage PDAC patients who went through curative resection and advanced PDAC patients who did not perform operation of curative intention. Because the endpoint of interest was OS, therefore, the survival length for early stage PDAC patients was defined as the time interval between curative resection and the date of death, whereas for advanced PDAC patients, the survival length was defined as the time interval between histopathological diagnosis and the date of death. Whether the included patients adopted adjuvant or palliative chemotherapy was determined through the sole or combined use of the following 5 most commonly used drugs for PDAC treatment in China: gemcitabine, nab-paclitaxel, 5-fluorouracil, irinotecan, and oxaliplatin. Prognostic significance of baseline and varying ALP was estimated simultaneously, for resected PDAC patients, baseline ALP was serum ALP results measured within 1 week (7 days) after curative resection, for advanced PDAC patients, baseline ALP was serum ALP tested also within 1 week (7 days) after confirmatory diagnosis. Dynamic ALP included all serum ALP tests measured within the whole survival period. In order to control for the confounding caused by end stage disease, ALP results measured within one month before the death were excluded during the analysis.

### Statistical analysis

Among all available statistical methods in estimating the effect of time-dependent covariate on survival endpoint, an extension to widely used Cox proportional hazards model which forwarded by Anderson and Gill was among the most flexible and powerful. A detailed description of this method can be found in Anderson and Gill’s work [17], and the analytical strategy of AG model in this study is provided in Appendix 1. Although time-dependent variables are ubiquitous in the field of cancer survival, the application of AG model is rare, only can be found in two previously published studies [18, 19].

In this study, we applied AG model to evaluate the influence of dynamic serum ALP which measured within the survival period on OS of PDAC patients. The association between baseline serum ALP and OS of PDAC was also explored by using Cox proportional hazards model. Serum albumin (ALB) is a nutritional indicator which can partly reflect the performance status of cancer patients. Considering that when estimating the association between ALP and OS of PDAC, performance status may be a potential confounder, we applied multivariate survival models which controlled for the influence of serum ALB. Besides, as an elevated serum ALP is a likely consequence of biliary obstruction [20], serum total bilirubin (TBIL), a much more sensitive indicator in diagnosing biliary obstruction, was also included into the multivariate model. Similarly, as serum ALP can indicate cholangitis, we also extracted results of leukocyte count, a commonly used diagnostic indicator of cholangitis [21].

With regard to the quantitative nature of ALP results, we further discussed the association between serum ALP and OS of PDAC by using the quartiles. Finally, an exhaustive sensitivity analysis was performed. We used cut-offs ranging from the 25th percentile (P_25_) to the 75th percentile (P_75_) to dichotomize ALP, and explored its influence on death hazard. The scales for serum ALP, ALB, TBIL and leukocyte count were units per liter (U/L), gram per liter (g/L), umol per liter (μmol/L), and 10^9^ per liter (10^9^/L).

All statistical analyses were executed by using SAS (version 9.3, SAS Institute Inc., Cary, NC.). The significance level for statistical tests or inference was defined as two-tailed probability less than 0.05.

## Results

### General characteristics of PDAC patients

Totally, we have successfully collected 537 histopathologically confirmed PDAC patients. The general characteristics of included patients were listed in Table 1. Among all patients, 96 were diagnosed as early stage disease and accepted curative resection subsequently, 441 were advanced patients. Altogether 274 patients were male, accounted for 51.02% of all. Sex distribution was comparable between resected and un-resected patients. Over 40% patients received adjuvant or palliative chemotherapy. Resected patients were observed younger age and longer survival length. Baseline serum ALP, ALB, TBIL, and leukocyte count were all statistically different between resected and un-resected patients.

**Table 1:** General characteristics of PDAC patients

Characteristics	Resected PDAC (Count = 96)	Un-resected PDAC (Count = 441)	Total (Count = 537)
	*N* (%)/Mean (Std.)	*N* (%)/Mean (Std.)	*N* (%)/Mean (Std.)
Age (Years)	61.17 (10.34)^†^	66.32 (10.84) ^†^	65.40 (10.92)
Sex (Male)	54 (56.25)	220 (49.89)	274 (51.02)
Chemotherapy (Yes)	40 (41.67)	185 (41.95)	225 (41.90)
Operation modality (Whipple)	54 (56.25)	–	–
Survival length (Days)	497.92 (256.86)^*^	306.10 (267.69)^*^	340.39 (275.55)
Baseline serum ALP (U/L)	111.18 (109.15) ^§^	237.96 (283.90) ^§^	215.29 (265.78)
Baseline serum ALB (g/L)	34.11 (5.51) ^§^	37.16 (6.71) ^§^	36.62 (6.61)
Baseline serum TBIL (μmol/L)	49.89 (61.64) ^§^	44.44 (90.16) ^§^	45.41 (85.74)
Baseline leukocyte count (10^9^/L)	12.99 (4.80) ^§^	7.30 (4.31) ^§^	8.32 (4.91)

^†^ *p* < 0.05 by *t* test

^*^ *p* < 0.05 by log-rank test

^§^*p* < 0.05 by Wilcoxon rank-sum test

### Baseline and dynamic ALP with OS of PDAC

**Fig. 1:**
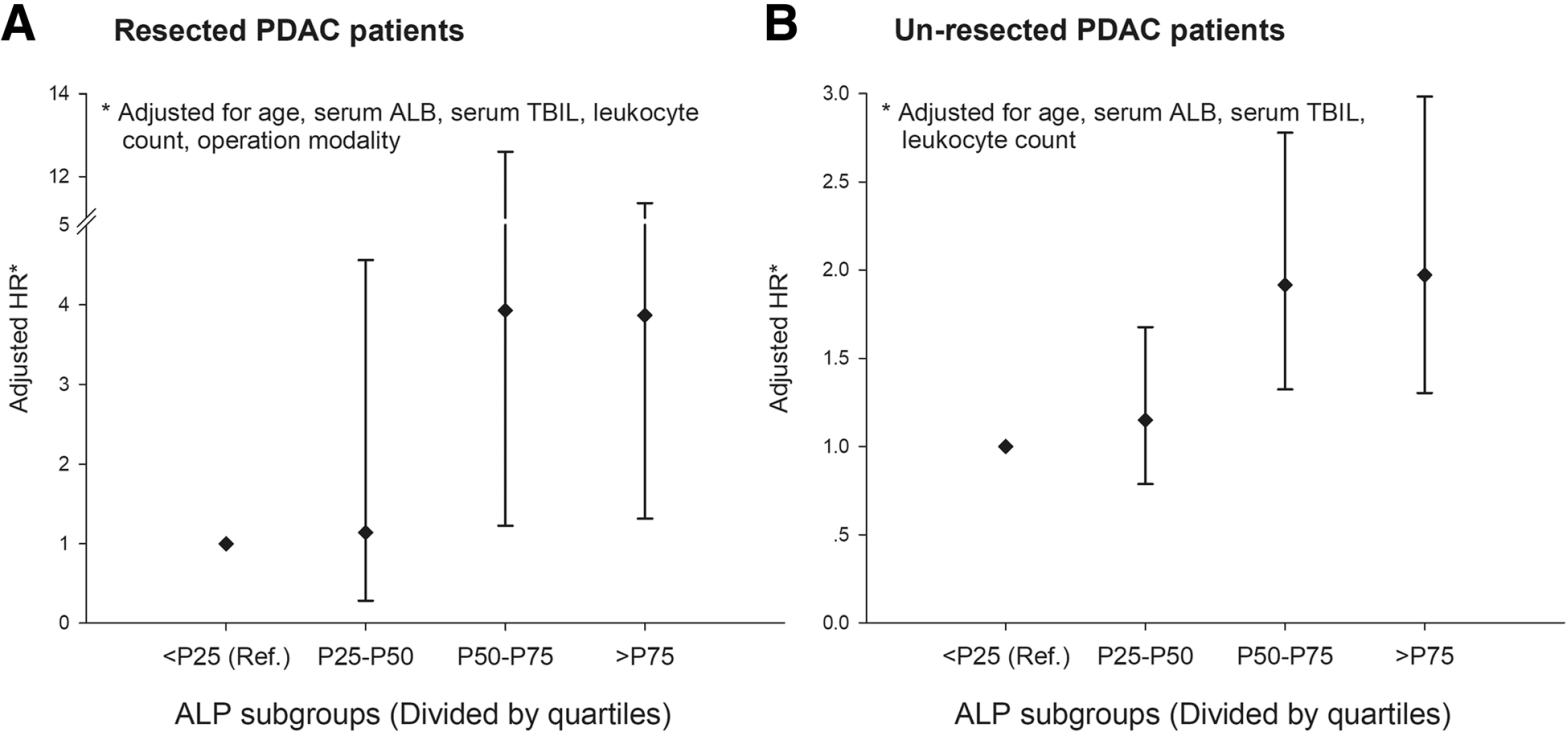
Varying serum ALP and OS of resected (**a**) and un-resected (**b**) PDAC patients

The influence of baseline and dynamic ALP on OS was evaluated separately in resected and un-resected PDAC patients, and the results were displayed in Table 2. While both multivariate Cox proportional hazards model and AG model revealed insignificant linear association between every 20 units increase of serum ALP and OS, the elevation of dynamic serum ALP was in general associated with deteriorated OS of PDAC: in resected patients, compared with the patients whose ALP results ranged within the first quartile (<P25), patients whose ALP measurements belonged to the second (P25-P50), the third (P50-P75), and the forth (>P75) quartiles were observed 1.14 (95% CI: 0.29–4.56), 3.93 (95% CI: 1.23–12.60), 3.87 (95% CI: 1.32–11.36) folds of death hazard; whereas in un-resected PDAC patients, the hazard ratios (HRs) were 1.15 (95% CI: 0.79–1.68), 1.92 (95% CI: 1.32–2.78), and 1.97 (95% CI: 1.30–2.98), respectively (Fig. 1).

**Table 2:** Baseline and dynamic serum ALP with the OS of PDAC patients by curative resection

Covariates		Cox proportional hazards model		AG model	
		Crude HR (95% CI)	Adjusted HR (95% CI)	Crude HR (95% CI)	Adjusted HR (95% CI)
Resected	Sex (Male)	1.17 (0.66, 2.06)		1.23 (0.71, 2.13)	
PDAC	Age (+ 5 years)	1.30 (1.11, 1.53)^**^	1.30 (1.11, 1.53)^**^	1.29 (1.13, 1.48)^**^	1.17 (0.95, 1.45)
	Chemotherapy (Yes)	0.80 (0.44, 1.43)		0.89 (0.52, 1.52)	
	Serum ALP (+ 20 U/L)	1.02 (0.97, 1.07)		1.09 (1.07, 1.11)^**^	1.09 (0.92, 1.29)
	Serum ALB (+ 5 g/L)	1.08 (0.82, 1.41)		0.52 (0.41, 0.65)^**^	0.66 (0.51, 0.87)
	Serum TBIL (+ 5 μmol/L)	1.01 (1.00, 1.03)		1.03 (1.01, 1.05)^**^	0.99 (0.96, 1.01)
	Leukocyte count (+ 1 10^9^/L)	0.98 (0.91, 1.04)		1.08 (1.04, 1.13)^**^	1.06 (1.02, 1.11)
	Serum ALP × Age	–		–	1.00 (0.99, 1.01)
Un-resected	Sex (Male)	0.92 (0.66, 1.28)		0.93 (0.76, 1.14)	
PDAC	Age (+ 5 years)	1.12 (1.03, 1.21)^**^	1.07 (0.99, 1.16)	1.09 (1.03, 1.14)^**^	1.11 (0.89, 1.37)
	Chemotherapy (Yes)	0.71 (0.51, 0.98)^**^	0.97 (0.65, 1.44)	0.69 (0.56, 0.84)^**^	1.52 (0.68, 3.40)
	Serum ALP (+ 20 U/L)	1.01 (1.00, 1.02)		1.03 (1.02, 1.03)^**^	0.99 (0.81, 1.22)
	Serum ALB (+ 5 g/L)	0.73 (0.64, 0.83)^**^	0.78 (0.66, 0.92)^**^	0.51 (0.46, 0.56)^**^	0.64 (0.48, 0.86)
	Serum TBIL (+ 5 μmol/L)	1.01 (1.00, 1.01)^*^	1.00 (0.99, 1.01)	1.03 (1.02, 1.03)^**^	0.98 (0.96, 1.01)
	Leukocyte count (+ 1 10^9^/L)	1.09 (1.03, 1.16)^**^	1.07 (1.01, 1.14)^**^	1.04 (1.02, 1.06)^**^	1.07 (1.02, 1.11)^**^
	Serum ALP × Age	–	–	–	1.01 (0.99, 1.02)
	Serum ALP × Chemotherapy	–	–	–	0.96 (0.91, 1.00)

^*^ *p* < 0.1 ^**^ *p* < 0.05

### Changing ALP cut-offs and the death hazard of PDAC

**Fig. 2:**
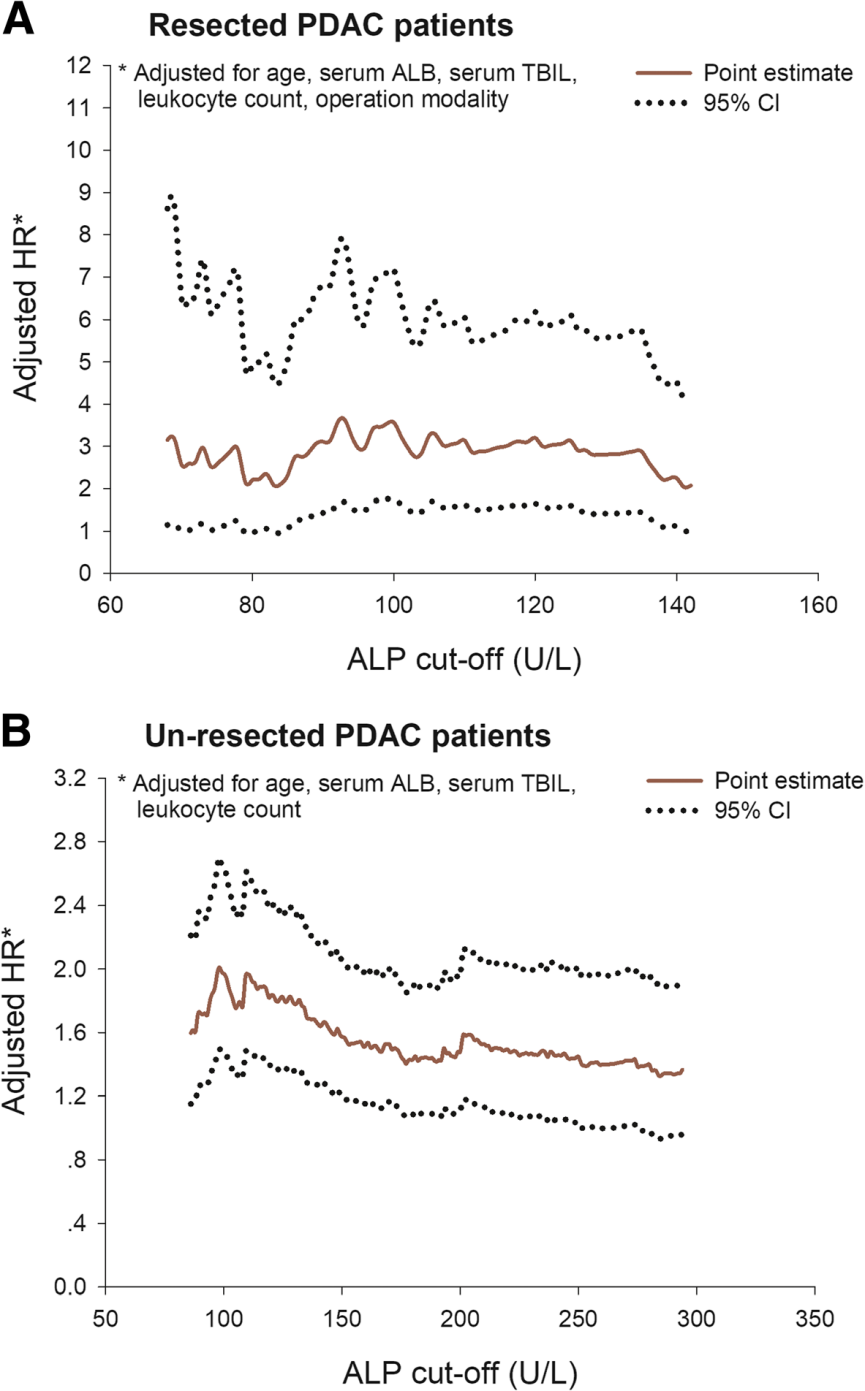
Changing cut-offs of serum ALP with the death hazard of resected (**a**) and un-resected (**b**) PDAC patients

For 96 resected PDAC patients, the P_25_ and P_75_ of their ALP measurements during the whole survival period were 68 U/L and 142 U/L, by applying the analytical strategy stated above, we dichotomized the patients by using ALP cut-offs ranging from 68 U/L to 142 U/L at every 1 U/L increment, and fitted a group of multivariate AG models subsequently. The adjusted HRs based on different ALP cut-offs were summarized in Fig. 2(a). For 454 un-resected PDAC patients, the P_25_ and P_75_ of ALP measurements were 86 U/L and 294 U/L. AG models were also fitted by using ALP cut-offs ranging from this interval at every 1 U/L increment. The HRs based on changing ALP cut-offs were depicted in Fig. 2(b).

For both resected and un-resected PDAC patients, changing ALP cut-offs did not overturn the general pattern of death hazard: no matter what specific cut-off was used, a higher level of ALP during the survival period was consistently associated with increased hazard of death. However, for different subgroups of patients, some distinctive features of association have also been observed. For example, an elevated ALP generally exhibited a much stronger influence on OS in resected PDAC patients. Nevertheless, sharper fluctuations in HRs were presented along with the change of ALP cut-offs among un-resected PDAC patients, and the HR culminated at ALP cut-offs near 100 U/L.

## Discussion

In this retrospective study, we explored the prognostic significance of varying ALP measured within the survival period in a large sample of PDAC patients by using dynamic AG model. Based on the analytical results, we found that, either in resected PDAC patients or in un-resected PDAC patients, especially among resected patients, an elevated ALP was generally associated with deteriorated OS. This finding is robust based on intensive sensitivity analysis by using a continuous range of ALP cut-offs. It suggests a possible prognostic role of ALP in PDAC survival.

A previously published study has found that an elevated ALP was prominently associated with lymph node involvement in resected esophageal cancer patients [22], thus, ALP level may be a sensitive indicator of tumor proliferation. As another type of solid malignant tumor, it is possible that among PC patients, especially among resected PC patients, an increased ALP can be associated with lymph node involvement, which may result in rapid relapse and progression of the disease. Another study with relevant laboratory findings further corroborated this suspicious pro-proliferation role of ALP at cell level [23]. The other possible explanation to this deleterious role of ALP in PDAC survival is that, ALP can be associated with occult metastasis in liver or bone tissue which cannot be identified through imaging examination, as suggested by the old evidence [24]. With this regard, an elevated ALP level among PDAC patients actually can indicate a more advanced stage of the disease.

Subsequently performed analysis revealed that, the elevation of ALP value was associated with more drastic increase in death hazard among resected PDAC patients. A natural suspicion is that, resection can be an ideal surrogate of cancer stage, as resected patients were usually in the early stage of the disease, and un-resected patients were diagnosed at the advanced stage of the disease. Therefore, it is possible that in un-resected advanced patients, the effect of ALP was masked by the rapid progression of the disease. Nevertheless, aside from this suspicion, plausible underlying mechanisms behind this finding should also be explored by future studies.

A very important finding of our study is that, when we using various cut-offs to dichotomize ALP, there was a distinctively different pattern in death hazard fluctuation: for resected PDAC patients, when ALP cut-offs ranging from 68 to 142 U/L, the HRs were mildly oscillating, whereas for un-resected PDAC patients, the HRs peaked around the ALP cut-off of 100 U/L. This observation probably suggests that, for PDAC patients of different disease stage, disparate cut-offs of ALP should be applied to identify patients with increased hazard. The most commonly used cut-off for serum ALP is no higher than 140 U/L [25]. However, our study results indicated that, this cut-off may not be suitable for PDAC patients. In resected early stage patients, even a cut-off as low as 68 U/L was associated with 3.15 folds of death hazard, which indicates that, for resected PDAC patients, the concept of ALP cut-off should be less stressed, lower level may predict better survival. However, because we only had 97 resected PDAC patients, with regard to statistical efficiency, cut-offs below 68 U/L were not analyzed, whether a preferable intervention cut-off exists for resected PDAC patients should also be discussed by future studies with expanded sample size. In un-resected advanced stage patients, an ALP level of higher than 100 U/L during the survival period can be associated with hiked hazard of death.

Although we have controlled for the possible prominent confounding caused by serum ALB, TBIL, and leukocyte count, residual confounding which caused by un-controlled confounders, like the concurrent colangitis of PC patients, other clinical heterogeneities, follow-up variation, will always exist. Aside from this, some other limitations of the current study should also be pointed out. First, we were unable to attain detailed tumor stage information of PDAC patients. However, curative operation information we used can be an ideal surrogate of tumor stage. Second, the PDAC patients we studied were exclusively Chinese, and the proportion of chemotherapy in Chinese PC patients is much lower than which in developed western countries [26, 27]. Therefore, caution should be made when extrapolating the results to other populations of higher chemotherapy administration proportions. Third, as we only included PC patients with complete information, the risk of selection bias may exist. Finally, differential follow-up and measuring frequency for different PC patients can also be a possible source of bias.

## Conclusions

In this study, by adopting dynamic AG survival model, we found that varying serum ALP which measured within the survival period was significantly associated with OS in a large group of PDAC patients. An elevated ALP was in general related to increased hazard of death among either resected or un-resecred patients, especially among resected patients. Our study results suggested that, serum ALP can be a meaningful prognostic index of PDAC. Future studies with larger sample size of resected PDAC patients and prospective design are warranted to validate our major findings. Besides, underlying mechanisms for this deleterious role of ALP in PDAC survival should also be explored.

## Data Availability

The datasets analyzed during the current study are not publicly available due to confidentiality agreement, but are available from the corresponding author on reasonable request.

## Appendix

### Details about Anderson-Gill counting process model (AG model)

Here we would like to explain how we analyze the “varying” values of alkaline phosphatase (ALP) by using AG model. We take one particular patient to elucidate the analytical process. However, when performing the analysis, we actually do the same thing to every included subject.

Suppose we have one PDAC patient, who is diagnosed as the advanced disease, cannot be performed curative resection, and his survival interval was the time length between histopathological diagnosis (time point A) and the date of death (time point D). During the whole survival period, suppose he performed 3 times of serum ALP test, at time points A, B and C.

Considering the varying nature of serum ALP, it is almost impossible that the test results of two neighboring time points will be exactly the same, so that means time point B is different from time point A, time point C is different from time point B. In order to adjust for this variation of ALP, AG model provides a straightforward solution which concentrates on the reconstruction of survival data into “counting process” style: we split the original observation into several sub-observations at the time points when this time-dependent variable fluctuates. In this example, compared to A, the test result of ALP at B changed, which means that, it will be more prudent to assume the test result of A presents the general status of ALP only for time interval AB, therefore we can treat AB as a sub-observation, and because of the subject did not present the outcome of interest (death) at time point B, so it is reasonable to define that for sub-observation AB, censoring occurred. Following the same logic, we can split the other two sub-observations BC and CD (for sub-observation CD, death occurred). For BC, we use ALP measured at B to represent the general status of ALP during this time interval, and for CD, ALP measured at C will represent the general status of ALP during this time interval.

Therefore, after this kind of “counting process” adjustment, instead of a single observation, we now have 3 sub-observations (AB, BC and CD). For these sub-observations, proportional hazard assumption of Cox model now held (because for each sub-observation, ALP result no longer varies, it is an essential assumption for using Cox model), and Cox model then can be used to analyze the adjusted data. However, only a little bit different from Cox model, when analyzing counting process adjusted survival data, AG model extra adjusts for inter-correlation of the sub-observations by using “Sandwich estimator”, for several sub-observations may come from a single patient.

## Abbreviations

ALB: Albumin
ALP: Alkaline phosphatase
HR: Hazard ratio
OS: Overall survival
PDAC: Pancreatic adenocarcinoma
TBIL: Total bilirubin
